# Prognostic significance of different molecular typing methods and immune status based on RNA sequencing in HR-positive and HER2-negative early-stage breast cancer

**DOI:** 10.1186/s12885-022-09656-4

**Published:** 2022-05-14

**Authors:** Xinyu Ren, Yu Song, Yanna zhang, Huanwen Wu, Longyun Chen, Junyi Pang, Liangrui Zhou, Songjie Shen, Zhiyong Liang

**Affiliations:** 1 Department of Pathology, Molecular Pathology Research Centre, Peking Union Medical College Hospital, Chinese Academy of Medical Science and Peking Union Medical College, No.1 Shuaifuyuan street, Dongcheng District, Beijing, China; 2 Department of Breast Surgery, Union Medical College Hospital, Chinese Academy of Medical Science and Peking Union Medical College, PekingBeijing, China

**Keywords:** HR-positive/HER2-negative breast cancer, Immune rescore, Intrinsic subtype, Risk of recurrence, Tumor-infiltrating lymphocyte

## Abstract

**Background:**

This study was conducted to evaluate the prognostic significance of different molecular typing methods and immune status based on RNA sequencing (RNA-seq) in hormone receptor (HR)-positive and human epidermal growth factor receptor 2 (HER2)-negative (HR + /HER2-) early-stage breast cancer and develop a modified immunohistochemistry (IHC)-based surrogate for intrinsic subtype analysis.

**Methods:**

The gene expression profiles of samples from 87 HR + /HER2- early-stage breast cancer patients were evaluated using the RNA-seq of Oncotype Dx recurrence score (RS), PAM50 risk of recurrence (ROR), and immune score. Intrinsic tumor subtypes were determined using both PAM50- and IHC-based detection of estrogen receptor, progesterone receptor, Ki-67, epidermal growth factor receptor, and cytokeratins 14 and 5/6. Prognostic variables were analyzed through Cox regression analysis of disease-free survival (DFS) and distant metastasis-free survival (DMFS).

**Results:**

Survival analysis showed that ROR better predicted recurrence and distant metastasis compared to RS (for DFS: ROR, *P* = 0.000; RS, *P* = 0.027; for DMFS, ROR, *P* = 0.047; RS, *P* = 0.621). Patients with HR + /HER2- early-stage breast cancer was classified into the luminal A, luminal B, HER2-enriched, and basal-like subtypes by PAM50. Basal-like subgroups showed the shortest DFS and DMFS. A modified IHC-based surrogate for intrinsic subtype analysis improved the concordance with PAM50 from 66.7% to 73.6%, particularly for basal-like subtype identification. High level of TILs and high expression of immune genes predicted poor prognosis. Multi-factor Cox analysis showed that IHC-based basal-like markers were the only independent factors affecting DMFS.

**Conclusions:**

Prognosis is better evaluated by PAM50 ROR in early-stage HR + /HER2- breast cancer and significantly differs among intrinsic subtypes. The modified IHC-based subtype can improve the basal-like subtype identification of PAM50. High immunity status and IHC-based basal-like markers are negative prognostic factors.

**Supplementary Information:**

The online version contains supplementary material available at 10.1186/s12885-022-09656-4.

## Background

Breast cancer is a heterogeneous disease from both the clinical and pathological perspectives [1, 2]. Although early detection is critical, some cases diagnosed in early stages may persist and recur locally, cause distant metastasis, or even result in mortality. In the last decade, determination of the tumor gene expression profiles has considerably improved the understanding of the biological heterogeneity of breast cancers [2, 3]. Analysis of gene expression panels, such as Oncotype DX and PAM50, can provide prognostic information beyond traditional assessments of the risk of breast cancers [4, 5]. Alternatively, the Oncotype DX panel (21 genes) calculates the recurrence score (RS), and the PAM50 (50 genes) provides the risk of recurrence (ROR) score, which also identifies intrinsic subtypes. Because of the limited use of molecular detection in Chinese patients, the abilities of Oncotype and PAM50 to accurately predict prognosis in patients with hormone receptor (HR)-positive human epidermal growth factor receptor 2 (HER2)-negative early breast cancer have not been compared.

Intrinsic molecular profiling provides additional clinically relevant insights compared to current pathology-based classifications. However, the technological complexity and high operating costs have limited its use in the clinic, leading to the development of immunohistochemistry (IHC)-based surrogates for identifying intrinsic subtypes of breast cancer (luminal A, luminal B, HER2-enriched, basal-like, and normal-like [6, 7]. IHC analysis of the expression of ER, progesterone receptor (PR), HER2, and the proliferation marker Ki-67 have been used to replace genotyping in clinical applications [8]. This pathology-based classification is well-established and used clinically to determine treatment modalities (endocrine therapy, chemotherapy, and/or targeted therapy) and patient inclusion in clinical trials. The discordance between IHC- and PAM50-based intrinsic subtypes was previously reported; however, the discordance in the survival data of patients with early-stage breast cancer has not been established [9, 10].

The immune status, particularly that of tumor-infiltrating lymphocytes (TILs), was recently identified as a useful marker for predicting prognosis and responses to neoadjuvant chemotherapy in patients with ER-positive breast cancer [11, 12]. The immune-specific gene expression patterns of TILs can be evaluated by quantifying differences in the abundance of multiple immune infiltrates in various solid tumors [13, 14]. However, the prognostic role of the immune status in HR-positive/HER2-negative (HR + /HER2-) early breast cancers and its relationship with different intrinsic subtypes are unclear.

This study was conducted to compare the prognosis prediction efficiency of two multi-gene expression panels and explore the prognostic effect of the immune status on HR + /HER2- early-stage breast cancer. Additionally, we improved the IHC-based surrogate method for predicting prognosis by incorporating the immune status into the model.

## Methods

### Patients

We recruited 87 patients with HR + /HER2- stage 1 breast cancer who underwent curative surgery at Peking Union Medical College Hospital (Beijing, China) between July 2012 and August 2017. The median age of the patients was 48.5 years (range, 30–78 years). The median follow-up duration was 60 months. Clinical information regarding age, TNM staging, disease recurrence, and death of all patients was collected for further analysis. The TNM stage was described according to the AJCC 8^th^ edition, and only patients with HR + /HER2- stage 1 breast cancer was included in the study. This study is approved by the ethics committee on Human Research of Peking Union Medical College Hospital (S-K1445).

### Histological analysis

Sections of formalin-fixed paraffin-embedded breast tumor tissues were re-examined by evaluating the results of hematoxylin and eosin (HE) staining to confirm the diagnosis. The expression of ER, PR, HER2, Ki-67, epidermal growth factor receptor (EGFR), cytokeratin (CK) 14, and CK5/6 was analyzed via IHC using a Ventana BenchMark XT automated slide stainer (Ventana Medical Systems, Inc., Tucson, AZ, USA) according to the manufacturer’s instructions. For each antibody, positive and negative control slides were included in each staining run. IHC slides were independently assessed by two qualified pathologists. The IHC experimental conditions for all antibodies and criteria for interpreting the results have been described previously [15]. Briefly, nuclear staining for ER and PR in > 1% of the tumor cells was considered as a positive result. The HER2 status was determined by either negative (0 or 1) or equivocal (2 +) HER2 staining in IHC, and the absence of *HER2* amplification was confirmed by fluorescence in situ hybridization. The expression of CK5/6, EGFR, P53, and Ki-67 was detected at the time of diagnosis. Basal markers (CK5/6, EGFR, and CK14) were considered as positive when > 1% of the tumor cells displayed plasma or membrane staining of the basal markers.

Stromal TILs were also evaluated through HE staining by two experienced pathologists following the guidelines for TIL assessment recommended by the International TILs Working Group [16]. The percentage of stromal TILs were divided into low and high levels based on the receiver operating characteristic (ROC) curve, using a cutoff value of 13.5%.

### Gene expression analysis

The tumor surface area was determined by HE staining before RNA extraction. RNA was extracted from five 10-μm formalin-fixed paraffin-embedded slides using an RNAstorm extraction kit (CD201, CELLDATA, Fremont, CA, USA) following the manufacturer’s instructions, and macro-dissection was performed to protect the normal breast tissue from contamination. A minimum of 100 ng of purified total RNA was used to measure the expression of 50 tumor-related genes, 17 immune-related genes, and 5 housekeeping genes by RNA-seq on the iSeq platform (Illumina, San Diego, CA, USA), and a FASTQ file was generated for each sample. To guarantee the integrity of any downstream analysis, the sequencing data were further analyzed only when samples with less than 30% of missing genes among all genes and total reads larger than 10,000 were observed. In each FASTQ file, raw counts were generated using the ShortRead package in R; after a single read, one sequence was mapped to the targeted regions of the human genome. Next, the raw counts of all samples were normalized based on the sizes of the transcripts and library. A gene expression matrix was generated by calculating the counts per million per sample using the edgeR Bioconductor software package. Finally, the data were log2-transformed and evaluated using the K-nearest neighbor method with the median centered and column standardized gene expression data.

### Calculation of Relapse (RS and ROR), intrinsic subtype analysis, and immunity evaluation

The RS was calculated using the Oncotype Dx panel, and the ROR score was calculated using the PAM50 panel [4]. The proliferation-weighted ROR score was calculated as described previously [7]. Patients were assigned to one of three risk groups (low, medium, or high) according to the RS and ROR-P scores, respectively as described before [4, 7].

The four intrinsic subtypes of breast cancer, namely luminal A, luminal B, HER2-enriched, and basal-like, were identified using the PAM50 predictor based on the expression profiles [7].

Moreover, an immune score was determined based on the expression levels of 17 immune genes as previously described [17]. Patients were grouped into one of two immune groups, “iweak” or “istrong,” based on the immune score values. A suitable cutoff value, 45.5, was determined using the ROC curve (Supplementary Fig S1). An immune score ≥ 45.5 was considered as “istrong,” whereas an immune score < 45.5 was considered as “iweak”.

### IHC-based surrogate of intrinsic subtypes

IHC-based analysis of intrinsic subtypes was performed according to the St. Gallen guidelines, which identified only two subtypes (luminal A and luminal B) [18]. Luminal A was defined as ER- and PR-positive, HER2-negative, and Ki-67 “low” (≤ 30%); luminal B was defined as ER-positive, HER2-negative, and either PR-negative/low (< 20%) or Ki-67 “high” (> 30%).

Modified IHC-based analysis of the intrinsic subtype was performed to identify basal-like subtypes. Basal-like cases confirmed by PAM50 were investigated to determine the IHC features of ER, PR, Ki-67, and basal markers (EGFR, CK14, and CK5/6). The criteria for identifying a basal-like subtype in this assay were as follows: any one of the basal markers was positive, Ki-67 was “high” (≥ 40%), and ER was “low” (≤ 10%).

### Statistical analysis

SPSS version 17.0 (SPSS. Inc., Chicago, IL, USA) was used for statistical analysis. Qualitative variables were compared using the chi-square test or Fisher’s exact test. The false discovery rate was applied to multiple testing corrections.

Disease-free survival (DFS) was defined as the time from surgery to the date of the first local or distant relapse. Distant metastasis-free survival (DMFS) was defined from the date of curative surgery to the date of distant metastasis or the last follow-up. Relapsed disease and metastasis were verified by diagnostic imaging and pathology during follow-up examinations. Three patients died during the follow-up period, of whom two died from the disease. Because the number of deaths was very small, they could not be evaluated by regression analysis; therefore, we only analyzed the relationship of factors with DFS and DMFS. Survival analyses were performed using Kaplan–Meier curves. Statistical significance was set at a two-tailed *p*-value < 0.05.

## Results

### Clinical and histological characteristics

Of the resected tumors, 85 (97.7%) were invasive breast carcinoma of no special type, two were invasive lobular carcinoma. Majority of tumors (76 of 87, 87.4%) were low or media histologic grades (grade 1 or 2). Local recurrence was observed in 26 (29.9%) patients, whereas distant metastasis was observed in 15 (17.2%) patients. The median DFS time and DMFS time were 52 months and 55 months, respectively. Three patients (3.4%) died during follow-up, two of these deaths were attributable to breast cancer (Table 1).

**Table 1:** Patient characteristics

**Characteristic**	N (%)
**Age (years)**	
≤ 40	23(26.4%)
41–50	27(31.1%)
> 50	37(42.5%)
**Histologic subtype**	
No special type	85(97.7%)
lobular	2(2.3%)
**Tumor size (cm)**	
≤ 0.5	1(1.1%)
> 0.5, ≤ 1	20(23.0%)
> 1, ≤ 2	66(75.9%)
**Histologic Grade**	
1	17(19.6%)
2	59(67.8%)
3	11(12.6%)
**Ki67**	
≤ 30%	16(18.4%)
> 30%	71(81.6%)
**P53**	
Positive	25(28.7%)
Negative	62(71.3%)
**Basal-like marker**	
Positive	12(13.8%)
Negative	75(86.2%)
**TILs**	
≤ 10%	65(74.7%)
11–59%	19(21.8%)
≥ 60%	3(3.5%)
**IHC surrogated subtype**	
Luminal A	61(32.2%)
Luminal B	26(67.8%)
**Immune score**	
Strong	43(49.4%)
Weak	44(50.6%)
**Chemotherapy**	
Yes	26(29.9%)
No	61(70.1%)
**Endocrine therapy**	
Yes	87(100%)
No	0(0%)
**Radiotherapy**	
Yes	25(28.7%)
No	62(71.3%)
**Recurrent status**	
Yes	26(29.9%)
No	61(70.1%)
**Distant metastasis**	
Yes	15(17.2%)
No	72(82.8%)
**Living status**	
Yes	84(96.6%)
No	3(3.4%)

### ROR by PAM50 and its comparison to RS by Oncotype Dx

**Fig. 1:**
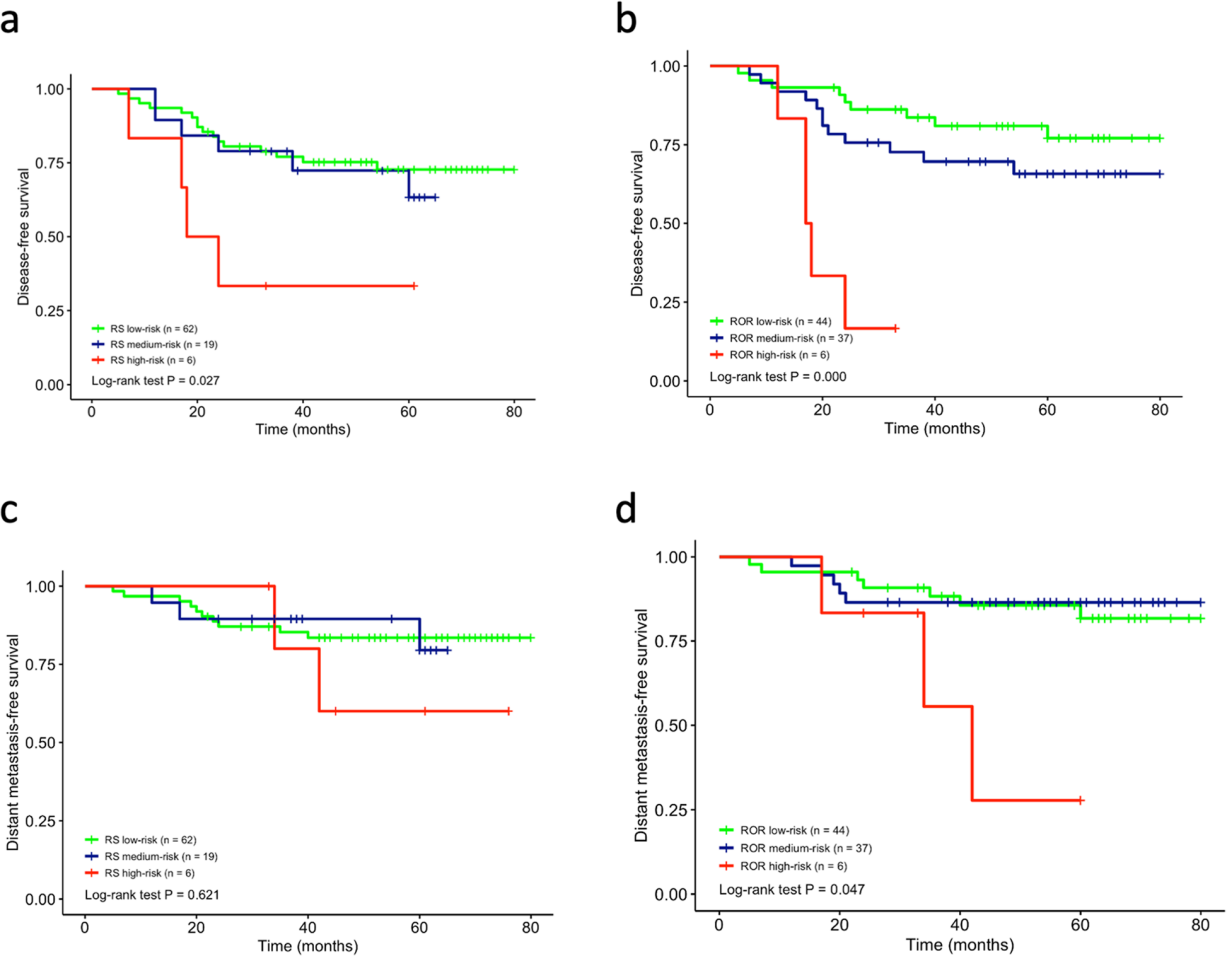
Survival analysis based on RS and ROR risk categories. Disease-free survival analysis by RS (**a**) and ROR (**b**); Distant metastasis-free survival analysis by RS (**c**) and ROR (**d**)

ROR and RS were categorized as low, medium, and high; the distributions of both methods are listed in Table 2. Kaplan–Meier survival analysis showed that the ROR measured by the two methods could predict actual recurrence (Fig. 1a; *P* = 0.027, Oncotype Dx; Fig. 1b; *P* = 0.0000, PAM50). The low, medium, and high ROR detected by PAM50 was consistent with the actual prognosis in the survival curve. The survival curve for low and medium risks overlapped with each other detected by Oncotype Dx (Fig. 1a). The ROR of PAM50 more accurately predicted DFS compared to Oncotype Dx (Fig. 1b). Neither method could effectively predict the DMFS of the low- and medium-risk groups, as the curves of the two groups overlapped in PAM50 (Fig. 1c and d). However, the ROR significantly differentiated the high-risk group from others when predicting DMFS (*P* = 0.621, Oncotype Dx; *P* = 0.047, PAM50) (Fig. 1c and d). The concordance of recurrence risks provided by the two methods was poor (κ = 0.194).

**Table 2:** Risk class based on ROR/RS

Subtype	Risk Class (ROR/RS)			*P*
	Low	Medium	High	
Luminal A (*n* = 62)	44/51	18/10	0/1	0.149
Luminal B (*n* = 17)	0/10	15/6	2/1	0.000
Basal-like (*n* = 6)	0/1	2/2	4/3	0.565
HER2-enriched (*n* = 2)	0/0	2/1	0/1	1.000
Total (*n* = 87)	44/62	37/19	6/6	0.012

### Distribution of intrinsic subtypes and its prognostic significance

**Fig. 2:**
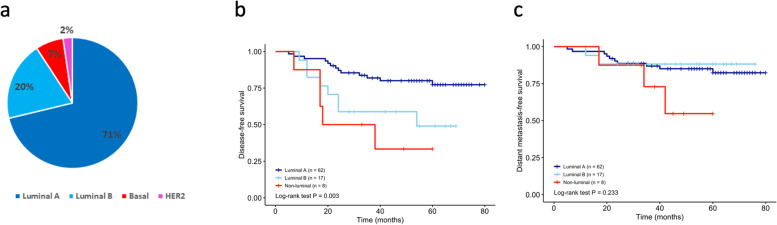
Intrinsic subtype distribution and prognosis evaluation. **a** Intrinsic subtypes classified by PAM50; Disease-free survival analysis by modified IHC surrogate (**b**); Distant metastasis-free survival analysis by PAM50 (**c**)

The following distributions of the intrinsic subtypes were detected by PAM50 analysis of the 87 analyzed breast tumors: 71% luminal A, 20% luminal B, 7% basal-like, and 2% HER2-enriched (Fig. 2a). Most HR + /HER2- breast cancers were of the luminal subtype. Six cases showed the intrinsic basal-like subtype and only 2 cases exhibited the HER2 enrichment subtype (too few for survival analysis). K-M analysis showed that the luminal subtypes had a significant longer DFS time than non-luminal type (Fig. 2b; *P* = 0.003) but the DMFS time did not show statistical significance (Fig. 2c; *P* = 0.233). According to the ROR of PAM50, the low-risk class consisted entirely of the luminal A subtypes, whereas the high-risk class comprised four basal-like and two luminal B subtypes (Table 2). Consistent with the ROR, the luminal A subtype displayed the longest DFS, and the basal-like subtype displayed the shortest DFS and DMFS (supplementary Fig. 2a; *P* = 0.02, Fig. 2b; *P* = 0.074). HER2-enriched subtype was excluded from the survival analysis due to the limited case number.

### Comparison of IHC-Based and PAM50 subtype analysis

The St. Gallen IHC-based intrinsic subtype analysis defined two subtypes of HR-positive breast cancer: 61 cases of luminal A and 26 cases of luminal B. In IHC-based subtype analysis, cases of luminal B subtype displayed a shorter but insignificant (*P* = 0.197) DFS compared to the luminal A cases; however, the survival curve of DMFS showed the opposite result (*P* = 0.394), with cases of luminal A displaying a shorter DMFS than those of luminal B, in contrast to the prognosis of the two subtypes reported by most studies [19].

**Fig. 3:**
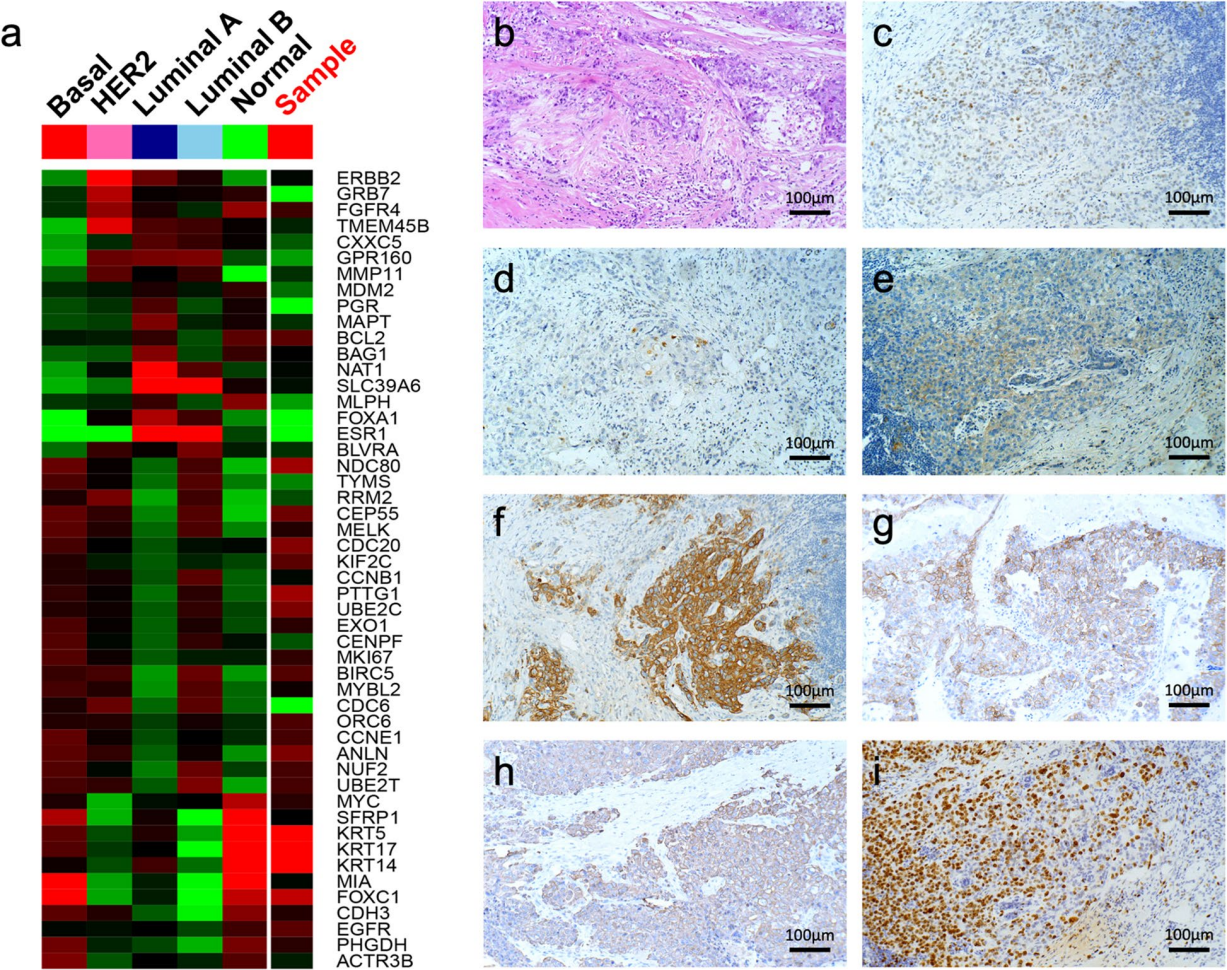
Basal-like subtype case defined by both PAM50 and modified IHC-based assay. PAM50 heatmap **a**; HE staining **b**; ER + , 10% **c**; PR + , 1% **d**; HER2, 1 + **e**; f. CK5/6 + **f**; EGFR + **g**; CK14 + **h**; Ki-67 index 80% **i**

**Fig. 4:**
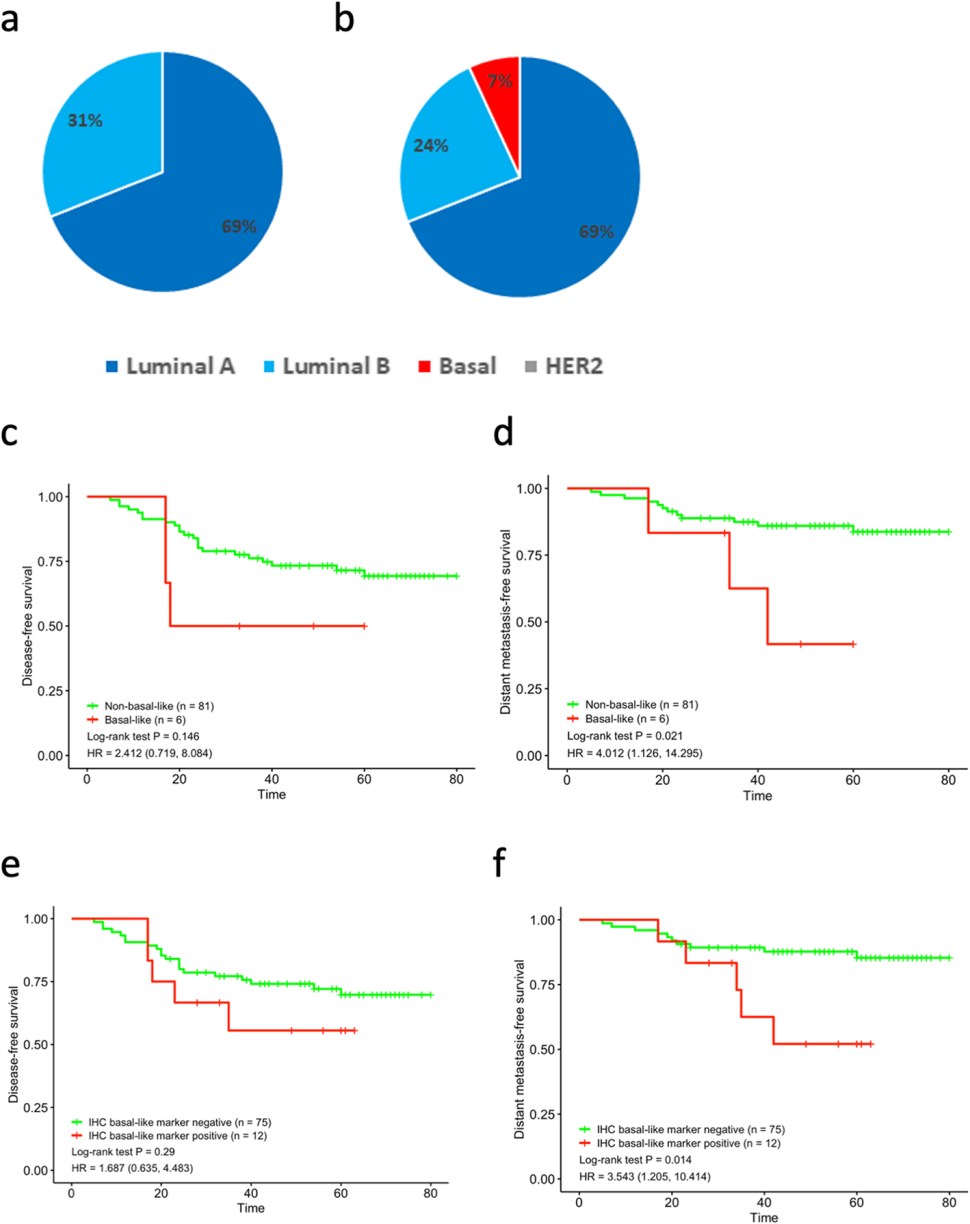
Prognosis evaluation of modified IHC surrogate and IHC basal-like marker. Intrinsic subtypes classified by St. Gallen IHC (**a**) and a modified IHC surrogate (**b**) Disease-free survival analysis by modified IHC surrogate (**c**); Distant metastasis-free survival analysis by modified IHC surrogate (**d**); Disease-free survival analysis of all cases expressing basal-like markers regardless of Ki-67 index (**e**); Distant metastasis-free survival analysis of all cases expressing basal-like markers regardless of Ki-67 index (**f**)

The concordance of subtype identification by PAM50 and the IHC-based method was poor (kappa = 0.246). We found that all PAM50-defined basal-like subtypes expressed basal-like markers, such as CK5/6, CK14, or EGFR (Tables 3, 4 and Fig. 3). The cases expressing both basal-like markers and showing a high Ki-67 growth index exhibited basal-like subtypes. Therefore, we modified the traditional IHC-based subtype by adding basal-like markers and an adjusted Ki-67 index, thereby enabling identification of all 6 cases (6.9%) of basal-like tumors defined by the PAM50 predictor, in addition to detecting 61 (70.1%) cases of luminal A and 20 (23.0%) cases of luminal B subtypes. Intrinsic subtypes identified by PAM50 were 66.7% and 73.6% concordant with those detected by IHC and modified IHC (Fig. 4a and b, respectively). Moreover, the modified IHC-based intrinsic subtypes recognized all cases of the molecular basal-like subtype with shorter DFS and DMFS times than those of non-basal-like subtype cases (for DFS, *P* = 0.146; for DMFS, *P* = 0.021; Fig. 4c and d). Further analysis revealed that all HR + /HER2- cases expressing basal-like markers had a worse prognosis, regardless of the Ki-67 index (for DFS, *P* = 0.29; for DMFS, *P* = 0.014; Fig. 4e and f).

**Table 3:** The clinicopathological characteristics of the molecular subtypes

Case No	IHC subtype	Molecular subtype	Basal-like marker	Ki67 index (%)	PR (%)	ER (%)	Grade	P53	Immune score	TILs	PAM50 recurrence risk	Recurrence/metastasis
1	Lum B	basal	positive	80	0	1	3	P	strong	high	high	Yes/m
2	Lum B	basal	positive	60	30	2	3	P	strong	high	med	no
3	Lum B	basal	positive	60	0	1	2	P	weak	1ow	med	no
4	Lum B	basal	positive	60	5	0	2	N	strong	high	high	Yes/m
5	Lum B	basal	positive	90	0	10	3	P	weak	1ow	high	no
6	Lum B	basal	positive	40	10	5	3	P	strong	high	high	Yes/m
7	Lum B	Lum A	positive	30	90	90	2	N	weak	1ow	med	no
8	LumA	Lum A	positive	6	60	80	2	N	weak	1ow	low	Yes/m
9	Lum B	Lum A	positive	10	0	95	1	N	weak	1ow	med	no
10	Lum B	Lum B	positive	20	95	95	2	N	weak	1ow	med	no
11	LumA	Lum A	positive	3	100	100	1	P	weak	1ow	low	no
12	LumA	Lum A	positive	10	30	60	1	N	weak	1ow	low	Yes/m

**Table 4:** Expression of different basal-like markers in basal-like marker positive cases

Cases No	Molecular subtype	IHC subtype	CK5/6	EGFR	CK14
1	Basal-like	Lum B	+	+	+
2	Basal-like	Lum B	+	+	-
3	Basal-like	Lum B	-	+	-
4	Basal-like	Lum B	+	+	+
5	Basal-like	Lum B	-	+	-
6	Basal-like	Lum B	+	+	-
7	Lum A	Lum B	-	+	-
8	Lum A	Lum A	-	+	-
9	Lum A	Lum B	-	+	-
10	Lum B	Lum B	-	+	-
11	Lum A	Lum A	-	+	-
12	Lum A	Lum A	-	+	-

### Impact of immune status on prognosis

**Fig. 5:**
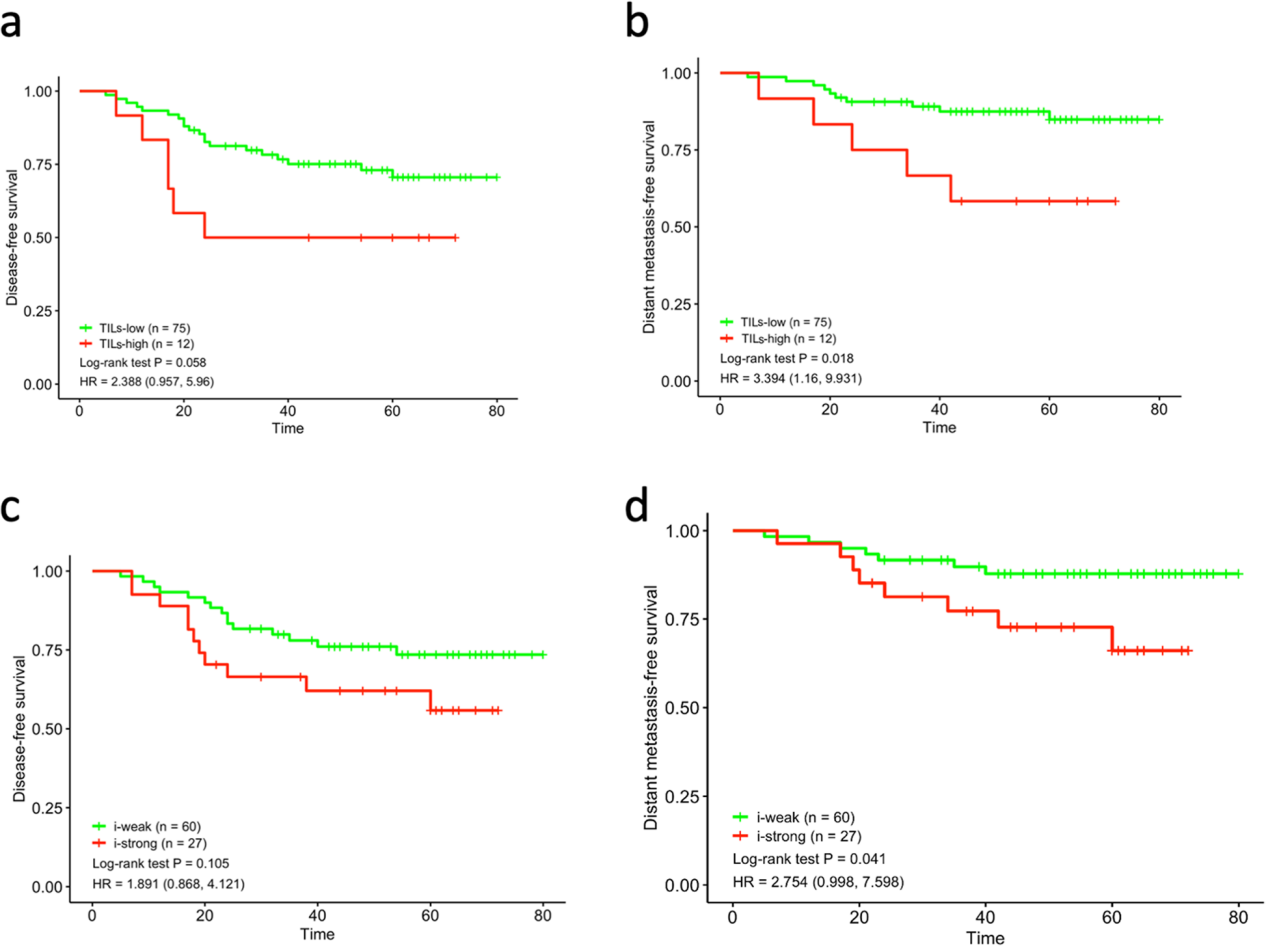
Immune status and prognosis. Disease-free survival analysis based on TILs subgroups (**a**) and immune score (**c**); Distant metastasis-free survival analysis based on TILs subgroups (**b**) and immune score (**d**)

The immune status was evaluated by examining the TILs and calculating the immune scores. For the relatively small number of cases in our cohort, we calculated the best cutoff value of TILs using the ROC curve as 13.5%. According to this cutoff value, there were 75 cases of low-level TILs and 12 cases of high-level TILs. High-level TILs cases showed relatively shorter DFS and DMFS, and the difference for DMFS was significant (*P* = 0.058 for DFS, *P* = 0.018 for DMFS, Fig. 5a and b). Additionally, an immune score cutoff value of 45.5 was obtained using the ROC curve to differentiate the cases into i-strong and i-weak groups. The i-strong group also showed a worse prognosis (*P* = 0.105 for DFS, *P* = 0.041 for DMFS, Fig. 5c and d).

Furthermore, the prognostic value of immune status differed according to the intrinsic molecular subtype in cases expressing basal-like markers. The three molecular basal-like cases with metastasis were istrong and had high levels of TILs. Both the non-molecular basal-like cases with metastasis were weak and had low levels of TILs.

### Univariate and multivariate analysis of factors affecting DFS

**Fig.6:**
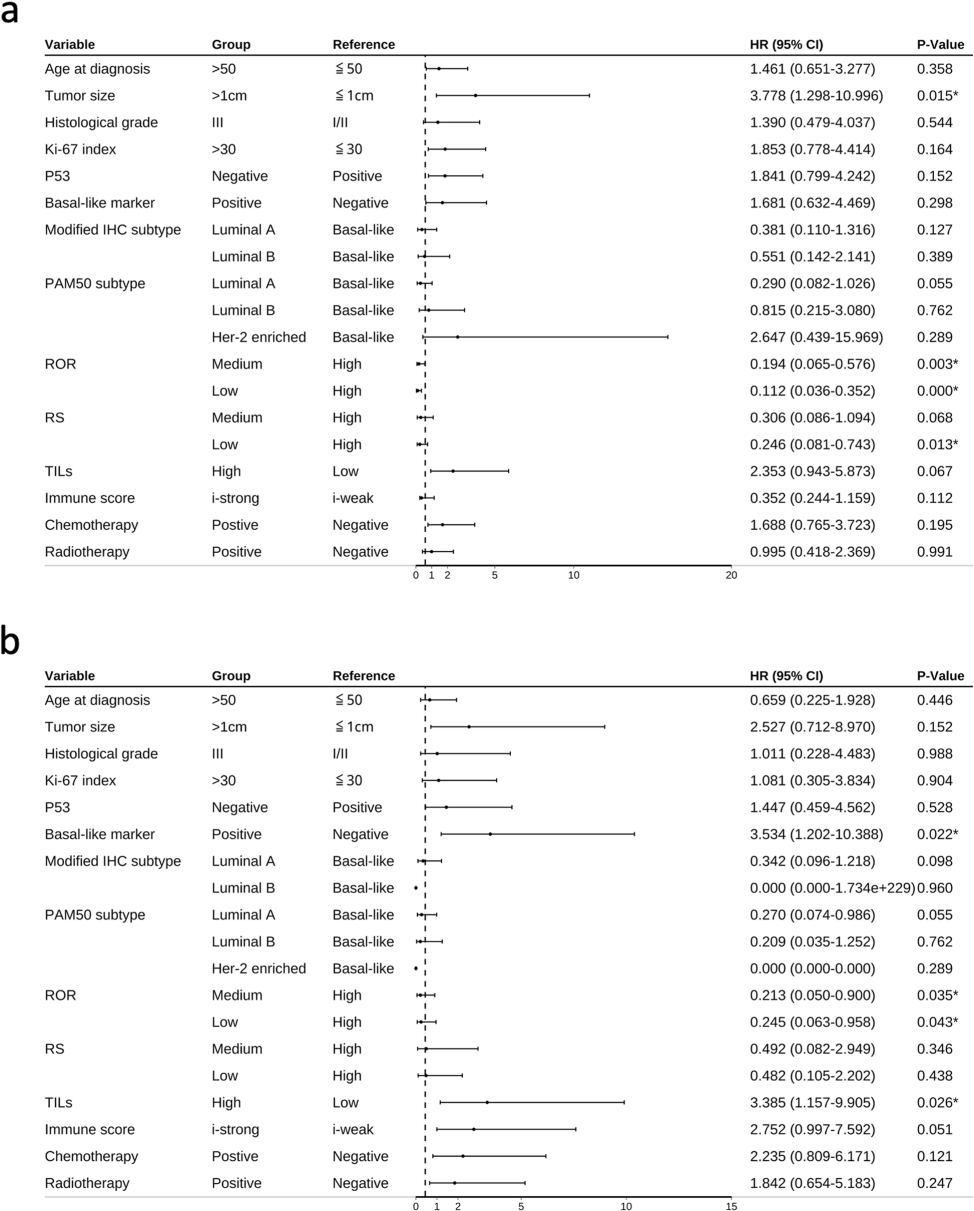
Forest plots of univariate analysis for DFS (**a**) and DMFS (**b**)

**Fig. 7:**
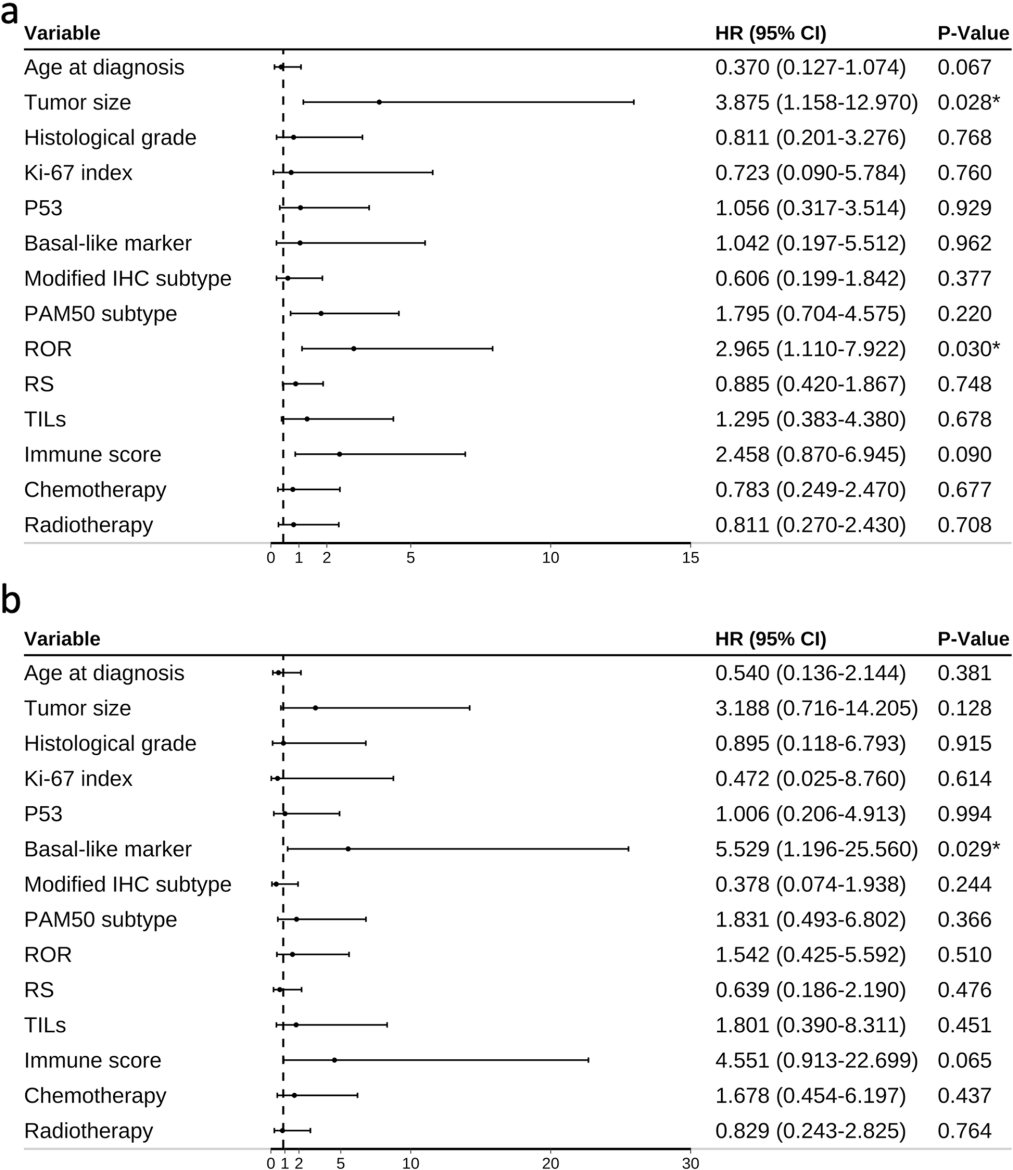
Forest plots of multivariate analysis for DFS (**a**) and DMFS (**b**)

Univariate analysis of 14 factors (age > 50 years, tumor size > 1 cm, histology high grade, Ki-67 index > 30%, IHC basal-like marker positive, modified IHC subtype, PAM50 molecular subtypes, ROR, RS, istrong, high level TILs infiltration, P53 positivity, chemotherapy, and radiotherapy) showed that four factors significantly affected DFS: tumor size, PAM50 subtypes, RS, and ROR (Table 5, Fig. 6a). Additionally, two factors significantly affected DMFS: IHC basal-like marker and high levels of TILs (Table 6, Fig. 6b). Multivariate analysis of the relevant factors revealed that a tumor size and ROR were independent factors affecting DFS (*P* = 0.028 and 0.030, respectively, Table 5, Fig. 7a). IHC basal-like markers positivity were the only independent factors affecting DFMS (*P* = 0.029, Table 6, Fig. 7b). Immune score was another factor that had great impact on DMFS in both univariate and multivariate analyses (*P* = 0.051 and 0.065 respectively, Table 6, Fig. 7). Other factors showed little effect on DFS and DMFS in both univariate and multivariate analyses.

**Table 5:** Univariate and multivariate Cox regression analysis of prognostic value of clinicopathological factors on tumor disease-free survival (DFS)

Variable (DFS)	Univariate cox regression analysis		Multivariate cox regression analysis	
	HR (95%CI)	*P*	HR (95%CI)	*P*
**Age at diagnosis**		0.358	0.370(0.127–1.074)	0.067
≦50	1			
> 50	1.461 (0.651–3.277)			
**Tumor size**		0.015^*^	3.875(1.158–12.970)	0.028^*^
≦1 cm	1			
> 1 cm	3.778 (1.298–10.996)			
**Histological grade**		0.544	0.811(0.201–3.276)	0.768
I/II	1			
III	1.390 (0.479–4.037)			
**Ki-67 index**		0.164	0.723(0.090–5.784)	0.760
≦30	1			
> 30	1.853 (0.778–4.414)			
**P53**		0.152	1.056(0.317–3.514)	0.929
Positive	1			
Negative	1.841 (0.799–4.242)			
**Basal-like marker**		0.298	1.042(0.197–5.512)	0.962
Negative	1			
Positive	1.681 (0.632–4.469)			
**Modified IHC subtype**		0.277	0.606(0.199–1.842)	0.377
Basal-like	1			
Luminal A	0.381(0.110–1.316)	0.127		
Luminal B	0.551(0.142–2.141)	0.389		
**PAM50 subtype**		0.006^*^	1.795(0.704–4.575)	0.220
Basal-like	1			
Luminal A	0.290(0.082–1.026)	0.055		
Luminal B	0.815(0.215–3.080)	0.762		
Her-2 enriched	2.647(0.439–15.969)	0.289		
**ROR**		0.001^*^	2.965(1.110–7.922)	0.030^*^
High	1			
Medium	0.194 (0.065–0.576)	0.003		
Low	0.112 (0.036–0.352)	0.000		
**RS**		0.045^*^	0.885(0.420–1.867)	0.748
High	1			
Medium	0.306 (0.086–1.094)	0.068		
Low	0.246 (0.081–0.743)	0.013		
**TILs**		0.067	1.295(0.383–4.380)	0.678
Low	1			
High	2.353 (0.943–5.873)			
**Immune score**		0.112	2.458(0.870–6.945)	0.090
i-weak	1			
i-strong	0.352 (0.244–1.159)			
**Chemotherapy**		0.195	0.783(0.249(2.470)	0.677
Negative	1			
Postive	1.688 (0.765–3.723)			
**Radiotherapy**		0.991	0.811(0.270–2.430)	0.708
Negative	1			
Positive	0.995(0.418–2.369)			

*P < 0.05

**Table 6:** Univariate and multivariate Cox regression analysis of prognostic value of clinicopathological factors on tumor distant metastasis-free survival (DMFS)

Variable (DMFS)	Univariate cox regression analysis		Multivariate cox regression analysis	
	HR (95%CI)	*P*	HR (95%CI)	*P*
**Age at diagnosis**		0.446	0.540(0.136–2.144)	0.381
≦50	1			
> 50	0.659 (0.225–1.928)			
**Tumor size**		0.152	3.188(0.716–14.205)	0.128
≦1 cm	1			
> 1 cm	2.527 (0.712–8.970)			
**Histological grade**		0.988	0.895(0.118–6.793)	0.915
I/II	1			
III	1.011 (0.228–4.483)			
**Ki-67 index**		0.904	0.472(0.025–8.760)	0.614
≦30	1			
> 30	1.081 (0.305–3.834)			
**P53**		0.528	1.006(0.206–4.913)	0.994
Positive	1			
Negative	1.447 (0.459–4.562)			
**Basal-like marker**		0.022^*^	5.529(1.196–25.560)	0.029^*^
Negative	1			
Positive	3.534 (1.202–10.388)			
**Modified IHC subtype**		0.253	0.378(0.074–1.938)	0.244
Basal-like	1			
Luminal A	0.342(0.096–1.218)	0.098		
Luminal B	0.000(0.000–1.734E + 229)	0.960		
**PAM50 subtype**		0.215	1.831(0.493–6.802)	0.366
Basal-like	1			
Luminal A	0.270(0.074–0.986)	0.055		
Luminal B	0.209(0.035–1.252)	0.762		
Her-2 enriched	0.000(0.000-)	0.289		
**ROR**		0.077	1.542(0.425–5.592)	0.510
High	1			
Medium	0.213 (0.050–0.900)	0.035		
Low	0.245 (0.063–0.958)	0.043		
**RS**		0.634	0.639(0.186–2.190)	0.476
High	1			
Medium	0.492 (0.082–2.949)	0.346		
Low	0.482 (0.105–2.202)	0.438		
**TILs**		0.026^*^	1.801(0.390–8.311)	0.451
Low	1			
High	3.385 (1.157–9.905)			
**Immune score**		0.051	4.551(0.913–22.699)	0.065
i-weak	1			
i-strong	2.752 (0.997–7.592)			
**Chemotherapy**		0.121	1.678(0.454–6.197)	0.437
Negative	1			
Positive	2.235 (0.809–6.171)			
**Radiotherapy**		0.247	0.829(0.243–2.825)	0.764
Negative	1			
Positive	1.842(0.654–5.183)			

*P < 0.05

## Discussion

Increasing evidence has shown that multi-gene expression panels used for breast cancer subtyping, such as Oncotype DX, MammaPrint, EndoPredict, Prosigna, and BCI, can provide prognostic information [18]. The clinical utility of these screening tools to guide treatment has been evaluated in several international studies [20, 21]. We compared the two most common multi-gene detection methods for ROR prediction in early-stage HR + /HER2- breast cancer. Our results showed that both the ROR of PAM50 and RS of Oncotype Dx analysis could be used to predict the prognosis of patients with HR + /HER2- breast cancer. However, the concordance of recurrence risks calculated for Oncotype Dx and PAM50 was poor. The ROR of PAM50 was found to more effective predict the risk of DFS and DMFS compared to using RS. Kaplan–Meier curves showed that RS could differentiate high RS levels from low and medium RS levels to predict DFS, although the curves for low and medium RS displayed overlap. The *P-*value of RS for predicting DMFS was not significant. ROR differentiated between the high, medium, and low levels of recurrence risk on the Kaplan–Meier curve and was more significant than RS for predicting DFS and DMFS. This result is consistent with previous research showing that ROR provides more prognostic information in endocrine-treated patients with ER-positive, lymph node-negative disease compared to RS, with better differentiation of intermediate- and high-risk groups [22]. ROR scores were reported to be more accurate for predicting the risk of distant metastasis compared to using RS among endocrine-treated postmenopausal women with ER-positive breast cancer [23]. Similarly, a study of 774 postmenopausal patients with ER-positive/HER2-negative breast cancer from the ATAC trial compared the prognostic values of six multi-gene signatures and demonstrated that ROR has a greater prognostic value for late distant metastasis compared to RS in patients with lymph node-negative breast cancer [24]. Compared to RS, ROR segregated more patients as medium-risk and fewer patients as low-risk. The accuracy of this categorization was confirmed by the marginally separated survival curve between the low- and medium-risk groups achieved using ROR rather than using RS. Of the 6 patients in the ROR-based high-risk class, 4 had the basal-like tumor subtype and 2 had the luminal B tumor subtype. The two cases of luminal B subtype had low Ki-67 levels (10% and 15%, respectively) and high ER expression, corresponding to the medium-risk group by the 21-gene method and low-risk group by the IHC surrogate method; therefore, neither group was administered chemotherapy. However, both patients experienced tumor relapse within 12–24 months. These patients may have benefitted from chemotherapy if they had been assigned to the high-risk class based on the ROR.

In addition to ROR, PAM50-based intrinsic molecular subtyping is commonly used to guide the clinical management of breast cancer treatments. Among HR + /HER2- early-stage breast cancers, most patients with luminal A breast cancer do not benefit from chemotherapy and only require endocrine therapy. However, luminal B breast cancer may require both chemotherapy and endocrine therapy. Furthermore, basal-like breast cancer may require stronger chemotherapy, and HER2-enriched cancers may benefit from targeted anti-HER2 therapy in combination with chemotherapy or endocrine therapy [18, 19, 25]. In our study sample, 9.2% (8/87) of patients with HR + /HER2- early-stage breast cancer in our cohort were non-luminal, which is within the previously reported range (8%–15%)[3]. Basal-like subtypes had the shortest DFS and DMFS times.

As for the poor prognosis of non-luminal breast cancers, it is important to differentiate between the non-luminal subtypes in HR + /HER2- patients. Although several international guidelines recommend using these multi-gene expression panels in the clinic to guide treatment [20, 21], the applicability of the PAM50 assay is limited in China. Surrogate tests based on IHC detection of ER, PR, HER2, and Ki-67 were developed to mimic PAM50 intrinsic subtyping and are more commonly used worldwide to guide the treatment of breast cancers [26]. However, Kim et al. reported a discordant rate of 38.4% between subtyping based on IHC and PAM50 in all breast cancers [10]. Using the St. Gallen IHC-based method for intrinsic subtyping (2015) in our study, only two subtypes, luminal A and luminal B, were detected, whereas no basal-like or HER2-enriched subtypes were identified. Additionally, poor concordance (66.7%) of intrinsic subtype identification between PAM50 and St. Gallen IHC-based surrogate subtyping was found in a previous study [3].

Unlike HR + /HER2- luminal breast cancer, the adjuvant therapy strategy in basal-like breast cancer is often very similar to that used in triple-negative breast cancers [3]. Identification of basal-like subtypes in HR + /HER2- breast cancers is important when a gene expression profiling assay cannot be performed. We found that all cases of basal-like intrinsic subtype defined by PAM50 expressed at least one of the basal-like markers and had a high Ki-67 index (≥ 40%) and low ER expression (≤ 10%). When the three basal markers (EGFR, cytokeratin CK14, and CK5/6) were added in conjunction with low ER expression and a high Ki-67 index in the IHC-based subtype assay, we identified all six PAM50 basal-like cases, and the concordance rate between the modified IHC-based intrinsic subtyping and those detected by PAM50 was increased to 73.6%.

Accumulating evidence has shown that TILs and immune-related gene signatures can be prognostic or predictive factors in breast cancer [27]. Although the ROR of PAM50 and its intrinsic molecular subtyping can provide valuable prognostic information, it does not include immune-related genes. The prognostic effect of the immune status is well-established in triple negative and HER2-enriched breast cancers [27, 28]. Moreover, TILs and immune scores are prognostic in ER-negative and highly proliferative breast cancers [17, 29]. However, little evidence exists regarding the role of immunity in HR + /HER2- breast cancers. We evaluated prognosis based on TILs and immune scores for all 87 cases and found that both TILs and the immune score were factors predicting worse prognosis (particularly a shorter DMFS) for HR + /HER2- early-stage breast cancers. Our results are similar to those of other studies that showed that high TILs were an adverse prognostic factor for survival in ER-positive/HER2-negative cases [30]. Blok et al. reported that patients with excess CD8-positive TILs did not benefit from exemestane treatment in early-stage breast cancers [31]. Most patients with HR + /HER2- early-stage breast cancer was administered adjuvant endocrine therapy, which may explain why those with high levels of TILs had shorter DFS and DMFS in our study. For breast cancer prognosis, the immune gene signatures expressed by TILs must be incorporated into current multi-gene panels to improve the prognostic value and guide treatment. Although many immune-related genes have emerged as prognostic or predictive biomarkers, a study of the expression level of 130 immune-related genes revealed a high level of heterogeneity in luminal breast tumors [32]. We tested a panel of 17 immune-related genes which had a strong predictive effect in triple-negative breast cancer in patients with HR-positive early-stage breast cancer [17]. Our results showed that the immune-related gene panel had prognostic value in HR + /HER2- breast cancer, particularly for DMFS. However, because of the small size of our cohort, further validation in larger cohorts is needed. We found that the effect of the immune status on the prognosis of HR + /HER2- cases varied according to the molecular subtype.

In conclusion, we found that prognosis significantly differed among intrinsic subtypes and was better evaluated by the PAM50 ROR than Oncotype DX RS in early HR + /HER2- breast cancer. Additionally, we found that a strong immune status negatively affected the prognosis of HR + /HER2- breast cancer and that immune-related gene signatures should be incorporated into current multi-gene tests to enhance the prognostic value of current molecular subtyping methods. When multi-gene based intrinsic subtype analysis is not available, a modified IHC-based subtyping assay for identification of basal-like subtype is recommended in HR + /HER2- patients. The impact of IHC basal-like marker expression on prognosis of HR + /HER2- breast cancer also should be pay attention to. Our study is limited mainly by the relatively small cohort sample size, which should be reinforced in a larger cohort in further studies.

## Supplementary Information

**Additional file 1:** **Supplementary Figure S1.** ROC curve of immune score. **Supplementary Figure S2.** Prognosis evaluation of intrinsic subtype of Luminal A, Luminal B and Basal-like subtype of PAM50.

**Additional file 2:** **Supplementary table 1.** Genes detected in this study. **Supplementary table 2.** The PAM50 molecular subtype of tumors from patients with local recurrence, distant metastasis, or death.

**Additional file 3.**

## Data Availability

The datasets generated and/or analysed during the current study are available from the corresponding author on reasonable request.

## Abbreviations

DFS: Disease-free survival
DMFS: Distant metastasis-free survival
EGFR: Epidermal growth factor receptor
HE: Hematoxylin and eosin
IHC: Immunohistochemical
ROC: Receiver operating characteristic
ROR: Risk of recurrence
RS: Recurrence score
TIL: Tumor-infiltrating lymphocyte
