# Supplementary material for: Prognostic significance of different molecular typing methods and immune status based on RNA sequencing in HR-positive and HER2-negative early-stage breast cancer
Source: BMC Cancer. 2022 May 14;22:548. doi: 10.1186/s12885-022-09656-4 (PMC9107692; doi:10.1186/s12885-022-09656-4)
Supplement: Supplementary file 1 — Additional file 1: Supplementary Figure S1. ROC curve of immune score. Supplementary Figure S2. Prognosis evaluation of intrinsic subtype of Luminal A, Luminal B and Basal-like subtype of PAM50. [file 12885_2022_9656_MOESM1_ESM.docx]

**Supplementary Figures**


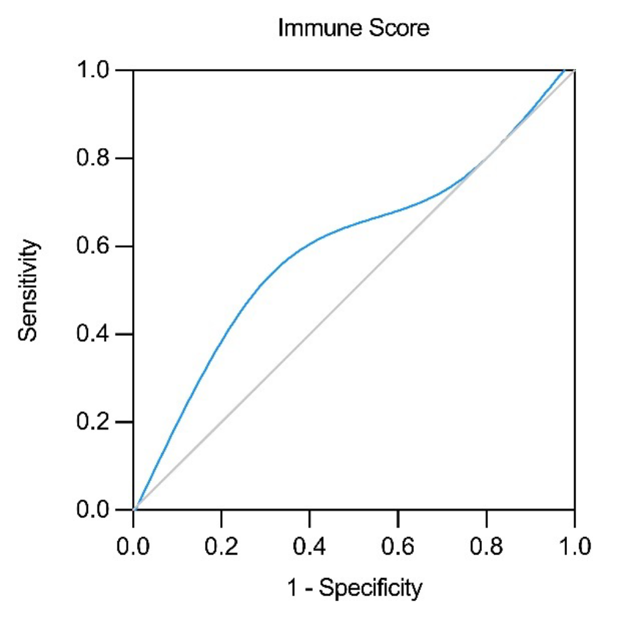


**Supplementary Figure S1. ROC curve of immune score.**


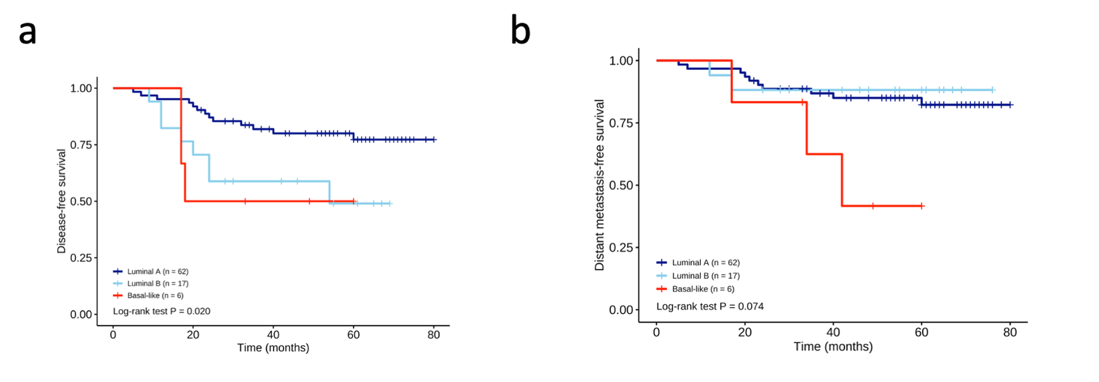


**Supplementary Figure S2. Prognosis evaluation of intrinsic subtype of Luminal A, Luminal B and Basal-like subtype of PAM50.** (a)Disease-free survival analysis. (b)Distant metastasis-free survival analysis.
