# Supplementary material for: Prognostic significance of different molecular typing methods and immune status based on RNA sequencing in HR-positive and HER2-negative early-stage breast cancer
Source: BMC Cancer. 2022 May 14;22:548. doi: 10.1186/s12885-022-09656-4 (PMC9107692; doi:10.1186/s12885-022-09656-4)
Supplement: Supplementary file 2 — Additional file 2: Supplementary table 1. Genes detected in this study. Supplementary table 2. The PAM50 molecular subtype of tumors from patients with local recurrence, distant metastasis, or death. [file 12885_2022_9656_MOESM2_ESM.docx]

**Supplementary materials**

Supplementary table 1. Genes detected in this study.

| Gene classification (n) | Gene symbols |
| --- | --- |
| Tumor-related genes (50) | ANLN, BIRC5, CCNB1, CCNE1, CDC20, CDC6, CENPF, CEP55, EXO1, KIF2C, MELK, MKI67, MYBL2, NDC80, NUF2, ORC6, PTTG1, RRM2, TYMS, UBE2C, UBE2T, ACTR3B, CDH3, EGFR, FOXC1, KRT14, KRT17, KRT5, MIA, MYC, PHGDH, SFRP1, ERBB2, GRB7, FGFR4, BAG1, BCL2, BLVRA, CXXC5, ESR1, FOXA1, GPR160, MAPT, MDM2, MLPH, MMP11, NAT1, PGR, SLC39A6, TMEM45B |
| Housekeeping genes (5) | GAPDH, GUSB, MRPL19, PSMC4, SF3A1 |
| Immune-related genes (17) | APOBEC3G, CCL5, CCR2, CD2, CD3D, CD52, CD53, CORO1A, CXCL9, GZMA, GZMK, HLA-DMA, HLA-DQA1, IL2RG, LCK, LYZ, PTPRC |

Supplementary table 2. The PAM50 molecular subtype of tumors from patients with local recurrence, distant metastasis, or death.

| Subtype | Recurrence state | | | Total |
| --- | --- | --- | --- | --- |
|  | LR | DM | Death |  |
| Luminal A (n=62) | 3 | 10 | 0 | 13 |
| Luminal B (n=17) | 6 | 1 | 1 | 8 |
| Basal-like (n=6) | 0 | 2 | 1 | 3 |
| HER2-enriched (n=2) | 2 | 0 | 0 | 2 |
| **Total** | 11 | 13 | 2 | 26 |
